# ERP Correlates of Verbal and Numerical Probabilities in Risky Choices: A Two-Stage Probability Processing View

**DOI:** 10.3389/fnhum.2015.00717

**Published:** 2016-01-21

**Authors:** Shu Li, Xue-Lei Du, Qi Li, Yan-Hua Xuan, Yun Wang, Li-Lin Rao

**Affiliations:** Key Laboratory of Behavioral Science, Institute of Psychology, Chinese Academy of SciencesBeijing, China

**Keywords:** risky choice, verbal probability, numerical probability, ERP

## Abstract

Two kinds of probability expressions, verbal and numerical, have been used to characterize the uncertainty that people face. However, the question of whether verbal and numerical probabilities are cognitively processed in a similar manner remains unresolved. From a levels-of-processing perspective, verbal and numerical probabilities may be processed differently during early sensory processing but similarly in later semantic-associated operations. This event-related potential (ERP) study investigated the neural processing of verbal and numerical probabilities in risky choices. The results showed that verbal probability and numerical probability elicited different N1 amplitudes but that verbal and numerical probabilities elicited similar N2 and P3 waveforms in response to different levels of probability (high to low). These results were consistent with a levels-of-processing framework and suggest some internal consistency between the cognitive processing of verbal and numerical probabilities in risky choices. Our findings shed light on possible mechanism underlying probability expression and may provide the neural evidence to support the translation of verbal to numerical probabilities (or vice versa).

## Introduction

We live in an uncertain world. Daily, people have to make decisions under uncertainty because they usually cannot control all the factors influencing the consequences of their decisions. Two kinds of probability expressions, verbal and numerical, have been used to characterize the uncertainty that we face. The verbal mode of probability expressions has a long history and has been considered the more natural system for processing probabilistic information (Zimmer, 1984). The numerical mode of probability expressions was first invented by legal scholars and later connected to the mathematical games of chance in the seventeenth century (Shafer, 1988). Numerical probability was suggested to be more accurate and to leave less room for subjective interpretation (Bonnefon and Villejoubert, 2006). The emergence of numerical probability enabled the development of Bayesian analysis.

Previous studies have shown that verbal and numerical probabilities differ in a number of ways (Wallsten et al., 1993). For example, numeric probability tends to elicit deliberate and rule-based reasoning from respondents, whereas verbal probability allows for more associative and intuitive thinking (Windschitl and Wells, 1996). There are two kinds of verbal probabilities denoting uncertainty: positive, suggesting the occurrence of a target outcome, and negative, drawing attention to its nonoccurrence (Teigen and Brun, 1999, 2000). Verbal probability distinguishes external attributions of uncertainty (disposition) from internal attributions of uncertainty (ignorance) (Kahneman and Tversky, 1982). People interpret verbal probability in a self-serving manner: verbal probabilities tend to be interpreted as denoting a higher or lower probability when they are used to describe the likelihood of pleasant or unpleasant events in one’s own future than when they are used to describe the likelihood of pleasant or unpleasant events in someone else’s future (Smits and Hoorens, 2005). Verbal probability seems to be the preferred way to express animate uncertainty, whereas numerical probability seems to be the preferred way to express inanimate uncertainty (Du et al., 2013).

However, behavioral analyses have indicated that people remain consistent between the two modes of response. By comparing numerical and non-numerical expressions of uncertainty, researchers have found that both types of expressions of uncertainty contain subjective magnitude information and that similar processes are involved in manipulating and comparing numerical and verbal terms (Jaffe-Katz et al., 1989). Vague meanings of probability (verbal probability) can be directly mapped into a 0–1 interval of probabilities (Wallsten et al., 1986; Reagan et al., 1989; Clark, 1990). In a study which asked participants to rate the attractiveness of lotteries based on previously equated verbal and numerical descriptors, both modes of judging uncertainty yielded reliable, internally consistent scales that demonstrated construct validity at the level of individual subjects (Budescu et al., 1988).

Behavioral evidence is indirect in nature and leaves unresolved the question of whether the cognitive processing of verbal and numerical probabilities is similar. The levels-of-processing framework suggests that an episodic memory trace may be thought of as an automatic by-product of operations carried out by the cognitive system and that the durability of the trace is a positive function of the depth of processing, where “depth” refers to greater degrees of semantic involvement (Craik and Tulving, 1975). Verbal and numerical probabilities are different modes of assessing uncertainty, but they are similar in that they both present uncertainty. According to the levels-of-processing concept, the processing of probability can be divided into two steps, early sensory processing and later semantic-associated operations. In the early sensory processing period, differences in the modes of probability expression (verbal or numerical) may affect the processing of uncertainty. In the later semantic-associated operations, the two different modes of probability expression could have attained some degree of consistency in their response at any given level of probability.

So far, a number of functional magnetic resonance (fMRI) studies have been conducted to investigate the neural basis of probability in risky decision making. Activation of the cortical medial prefrontal cortex, lateral prefrontal cortex, posterior parietal cortex, and insular cortices have been reported to be correlated with probability (Rogers et al., 1999; Paulus et al., 2003; Huettel et al., 2005, 2006; Knutson et al., 2005; Weber and Huettel, 2008; Smith et al., 2009; Mohr et al., 2010). However, due to the limited time resolution, fMRI technique is unable to distinguish two stages of probability processing if the signal is rapidly decaying over time (Kiefer, 2005).

In contrast, the high time resolution of the event-related potential (ERP) technique in cognitive neuroscience allows scientists to observe human brain activity that reflects specific cognitive processes over time (Pirtošek et al., 2009). Thus, in the present study we investigated the neural processing of verbal and numerical probabilities by exploiting the high temporal resolution of ERP recordings.

Studies in human subjects have shown that ERPs in the frontocentral regions of the scalp elicited more negative ERP deflections in high-risk situations than in low-risk ones in a 300–500 ms time window (Yang et al., 2007; Yang and Zhang, 2011). Using the Iowa Gambling Task, researchers found that high impulsive decision makers had a larger P3 than low impulsive decision makers (Martin and Potts, 2009). A recent study used magnetoencephalography to investigate the brain activity related to numerical probability and value information in decision-making (Steffen et al., 2011). They found that value information differentially affected detectable cortical responses in less than 150 ms, whereas activity did not vary as a function of probability information until 215 ms. Value and probability manipulations also differed in the extent of their anatomical and temporal impact: whereas the activity sensitive to value was confined to the bilateral temporoparietal region of interest (ROI) and ended quickly (230 ms), the activity sensitive to probability was more sustained and involved a cascade of three ROIs, spreading from the temporoparietal to the frontotemporal to the frontal ROI in about 100 ms. No value-probability interactions were observed. In combination, these findings indicate that value and probability play distinct roles in the first few 100 ms of processing, that they involve somewhat different parts of the brain at different times, and that they contribute additively rather than interactively to decision-making, at least of the sort assessed in the present task.

The existing ERP data about the time course of probability processing has been largely inconclusive but suggests that probability processing during risky decision-making may largely be carried out after 200 ms. Similarly, previous works on levels-of-processing framework have indicated that the representation mode affected the early sensory processing period about 100 ms after onset of stimulus and that the semantic meaning affected the later semantic-associated period about 200 ms after onset of stimulus (Dehaene, 1996). Thus, if similar processes are employed in manipulating and comparing numerical and verbal terms, we could expect that the representation mode of probability would affect the early period (100 ms) and that the level of probability would affect the later period (200 ms and later), independent of the representation mode.

In addition, according to the additive factors concept (Sternberg, 1969), which argued that if two factors affected different and independent stages of processing then the effect of varying one factor must be the same regardless of the level of the second factor, we hypothesized that if the processing of probability could be divided into two stages, there should be no significant interaction effect between representation mode and level of probability on the behavioral measures [i.e., percentage of risky choices and reaction time (RT)].

## Materials and Methods

### Participants

Twenty-five healthy undergraduate and graduate students (13 male, age = 21.9 ± 1.31) from China Agricultural University and Beijing Forestry University took part in the experiments. They were healthy with no past history of psychiatric or neurological disease and had normal or corrected-to-normal vision. Each participant provided written informed consent before the experiment. The study was approved by the Institutional Review Board of the Institute of Psychology, the Chinese Academy of Sciences.

### Materials

The participants were asked to choose between a certain option and a risky option. The certain option always yielded a sure outcome (CNY 10). The risky option offered a probability of a larger reward (CNY 20, 30, or 40). There were three levels of probability: low, medium, and high. At each level of probability, the outcomes for the risky option were fixed (CNY 20, 30, or 40). Two kinds of probability expressions, verbal and numerical, were used to characterize the probability. According to the work reported by Mohr et al. (2010), the mean numerical equivalents assigned for Chinese verbal probabilistic expressions of the low possibility, medium possibility, and high possibility were 20.41%, 52.52%, and 78.22%. Here, in the numerical probability expression, the three levels of probability were 20%, 50%, and 80%. In the verbal probability expression, the corresponding three levels of probability were low possibility, medium possibility, and high possibility.

### Procedure

**Figure 1:**
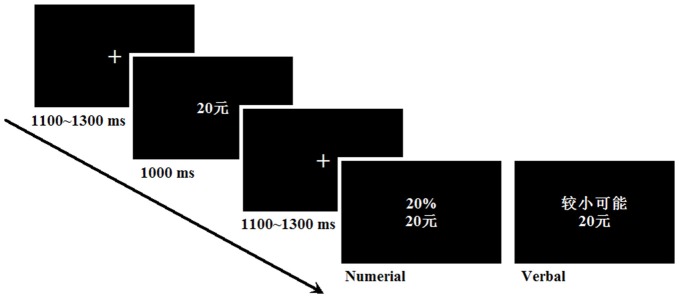
**Experimental design with an example of the sequence and timing of stimuli in a typical trial**.

Figure 1 shows the task process. Because the certain option was always CNY 10 in all trials, only the risky option was displayed on the screen to prevent interfering stimuli from affecting the perception of probability. In each trial, the participants were presented with a risky option and had to decide whether to accept or reject the risky option. If they rejected it, the participants would definitely receive CNY 10.

A single trial consisted of the following sequence: initially, a fixation was displayed in the center of the screen for 1100–1300 ms, followed by the presentation of the corresponding outcome of the risky option for 1000 ms; another fixation was then displayed for 1100–1300 ms, followed by the presentation of the corresponding probability of that risky option. Participants were asked to decide whether to accept or reject the risky option by pressing one of two buttons on a keyboard. The participants completed 486 trials (81 trials in each condition), divided into seven blocks of 72 or 54 trials each. The participants were informed that they would be paid CNY 50 for participating. To further incentivize their cooperation, they were also told that at the end of the experiment, one choice would be randomly selected to be played for real. Each participant was paid CNY 50–90 (~8–15 US dollars) for participating in the experiment.

The participants were instructed to keep their eyes focused on the center of the screen and to avoid unnecessary body and eye movements. The outcome stimuli were 2.4 cm in width and 0.8 cm in height. The risky option stimuli were 2.4 cm in width and 2.0 cm in height. The stimuli were presented in white against a black background at a distance of 70 cm, subtending a visual angle smaller than 5°. Eighteen practice trials were run in the first session to familiarize the participants with the task. The E-prime software was used to generate the visually presented problems (trials).

### Electrophysiological Recordings

We used a 64-channel Neuroscan to explore the specific cortical activity of electroencephalogram (EEG) signal. The EEG was recorded from 64 scalp sites according to the International 10–20 system using Ag/AgCl electrodes. Vertical and horizontal ocular movements were also recorded. Eye blinks were recorded from left supraorbital and infraorbital electrodes. The horizontal electrooculogram was recorded from electrodes placed 1.5 cm lateral to the left and right external canthi. All electrode recordings were referenced to an electrode placed on the left mastoid, and the impedance was maintained below 5 kΩ. The EEG was recorded using a band-pass of 0.1–100 Hz and digitized at a sampling rate of 500 Hz. The EEG data were re-referenced off-line to linked mastoid electrodes by subtracting from each data sample recorded at each channel one-half the activity recorded at the right mastoid. EEG epochs of 1000 ms (with a 100 ms prestimulus baseline) were extracted off-line for stimulus-locked ERPs when the risky options were presented. The data were baseline-corrected by subtracting from each sample the average activity of that channel during the baseline period. Trials with a voltage exceeding ±100 μV, relative to the 100 ms baseline, at any electrode were excluded from analysis, as were trials with artifacts in the electroocculograph channels. After excluding the trials that contained electrical artifacts, at least 71 trials remained in each condition for each participant.

### Data Analysis

#### Behavioral Data

The RTs were examined using 2 (probability expressions: verbal, numerical) × 3 (level of probability: low, medium, high) using repeated measures ANOVAs. The percentages of risky choices were examined by 2 (probability expressions: verbal, numerical) × 3 (level of probability: low, medium, high) using repeated measures ANOVAs.

#### ERP Data

Because the midline electrodes (Fz, FCz, Cz, CPz, Pz, POz, and Oz) have been reported to be related to risky decision making (Yang et al., 2007; Yang and Zhang, 2011), the ERP data were analyzed by performing 2 (probability expressions) × 3 (level of probability) × 7 (electrode) repeated-measures ANOVAs on the ERPs at seven electrodes. Visual inspection of the waveforms suggested a difference in the N1, N2, and P3 amplitudes between the conditions. The ERP data were analyzed by computing the mean amplitude in the 70–130 ms (N1), 250–330 ms (N2), and 340–550 ms (P3) time windows after the probability presentation. Mauchly’s test was used to test the sphericity assumption of the repeated measures ANOVA, and the Greenhouse-Geisser correction was used in cases where violations of sphericity were found. All statistical tests were two-sided and had an alpha level of 0.05.

## Results

### Behavioral Results

Table 1 shows the RT results and the percentage of risky choices under each condition. The 2 (probability expression) × 3 (level of probability) repeated measures ANOVAs conducted on the RT revealed a significant main effect of probability expression, *F*_(1,24)_ = 25.55, *p* < 0.001, *η*^2^ = 0.52; and a significant main effect of level of probability, *F*_(2,48)_ = 10.04, *p* = 0.001, *η*^2^ = 0.30. The interaction was not significant. *Post hoc* LSD analyses revealed that participants took a longer time in the verbal probability condition than in the numerical probability condition (*p* < 0.001). The RT in the high level of probability condition was significantly shorter than in the low (*p* < 0.001) and medium (*p* = 0.003) levels of probability, with no significant difference between the latter two conditions (*p* = 0.92).

**Table 1:** **Mean reaction time and the percentage of risky choices under each condition (*N* = 25)**.

Presentation mode	Level of probability	RT (ms) (*SD*)	Percentage of risky choice (*SD*)
Verbal	Low	748.3 (277.0)	0.14 (0.19)
	Medium	739.5 (314.4)	0.76 (0.26)
	High	663.2 (237.9)	0.92 (0.19)
	Low	708.7 (240.2)	0.13 (0.22)
Numerical	Medium	712.4 (325.0)	0.76 (0.26)
	High	612.7 (215.3)	0.99 (0.02)

A 2 (probability expression) × 3 (level of probability) repeated measures ANOVAs conducted on the percentage of risky choices revealed a significant main effect of level of probability, *F*_(2,48)_ = 192.33, *p* < 0.001, *η*^2^ = 0.89. No other significant effect was found. *Post hoc* LSD analyses revealed that the participants chose the least risky options in the low-possibility condition and the highest in the high possibility condition with the medium possibility intermediate between these (*p*s < 0.001).

### ERP Results

#### N1 Component (70–130 ms)

**Figure 2:**
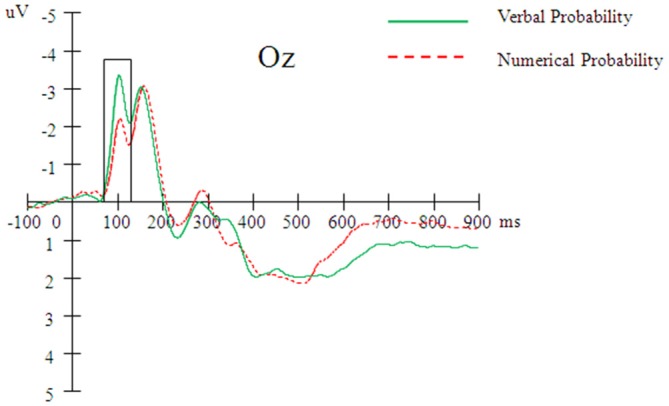
**Averaged event-related potential (ERP) waveforms (*n* = 25) elicited by verbal and numerical probability at the Oz electrode**.

A 2 (probability expression) × 3 (level of probability) × 7 (electrode) repeated measures ANOVA revealed a significant main effect of electrode, *F*_(6,144)_ = 7.61, *p* < 0.001, *η*^2^ = 0.24. A *post hoc* LSD analysis revealed the N1 amplitude at Oz to be greater than that at any other electrode (*p*s < 0.01). The ANOVA also revealed a significant interaction effect between electrode and probability expression, *F*_(6,144)_ = 12.77, *p* < 0.001, *η*^2^ = 0.35. A simple effect analysis indicated that the N1 amplitude elicited by the verbal probability was greater than that elicited by the numerical probability at the Oz electrode (*p* = 0.001; Figure 2), but the opposite pattern was observed at the Fz electrode (*p* = 0.03). No other effect was significant.

#### N2 Component (250–330 ms)

**Figure 3:**
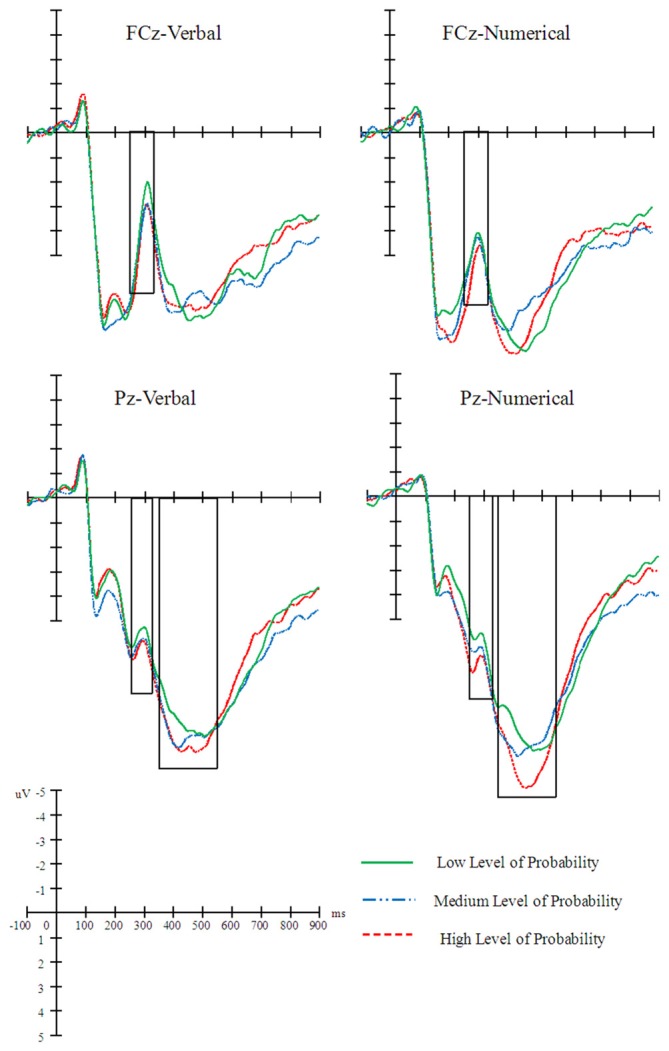
**Averaged ERP waveforms (*n* = 25) elicited by the level of probability in the verbal and numerical probability conditions at the FCz and Pz electrodes**.

A 2 (probability expression) × 3 (level of probability) × 7 (electrode) repeated measures ANOVA revealed a significant main effect of level of probability, *F*_(2,48)_ = 5.23, *p* = 0.01, *η*^2^ = 0.18. A *post hoc* LSD analysis revealed that the N2 amplitude elicited by the low level of probability was greater than that elicited by the high level of probability (*p* = 0.001). Repeated measures ANOVA also revealed a significant main effect of electrode, *F*_(6,144)_ = 17.45, *p* < 0.001, *η*^2^ = 0.42. A *post hoc* LSD analysis showed that the N2 amplitude was greater at the Oz electrode than at the other electrodes (*p*s < 0.01) and that the N2 amplitudes were smaller at the Pz and CPz electrodes than at other electrodes (*p*s < 0.05). In addition, the interaction between electrode and probability expression was significant, *F*_(6,144)_ = 6.22, *p* < 0.001, *η*^2^ = 0.21. A simple effect analysis revealed that the N2 amplitude elicited by the verbal probability was more negative than the amplitude elicited by the numerical probability at the FCz, Cz, and CPz electrodes (*p*s < 0.05; Figure 3). No other effect was significant.

#### P3 Component (340–550 ms)

A 2 (probability expression) × 3 (level of probability) × 7 (electrode) repeated measures ANOVA revealed a significant interaction effect between electrode and level of probability, *F*_(12,288)_ = 3.49, *p* < 0.001, *η*^2^ = 0.13. A simple effect analysis revealed that the amplitude elicited by the high level of probability was more positive than that elicited by the low level of probability at the Pz and POz electrodes (*p*s < 0.05). A repeated measures ANOVA also revealed a significant main effect of electrode, *F*_(6,144)_ = 52.97, *p* < 0.001, *η*^2^ = 0.69. A *post hoc* LSD analysis revealed that the amplitude at the Oz electrode was smaller than at the other electrodes (*p*s < 0.001) and that the amplitudes at the Pz and CPz electrodes were greater than the ones at other electrodes (*p*s < 0.01). The main effect of probability expression was significant, *F*_(1,24)_ = 6.42, *p* = 0.02, *η*^2^ = 0.21. A *post hoc* LSD analysis revealed that the amplitude elicited by numerical probability was more positive than that elicited by verbal probability. The interaction between electrode and probability expression was significant, *F*_(6,144)_ = 6.01, *p* < 0.001, *η*^2^ = 0.13. A simple effect analysis indicated that the amplitude elicited by numerical probability was more positive than that elicited by verbal probability at the Fz, FCz, Cz, CPz, and Pz electrodes (*p*s < 0.05; Figure 3).

**Figure 4:**
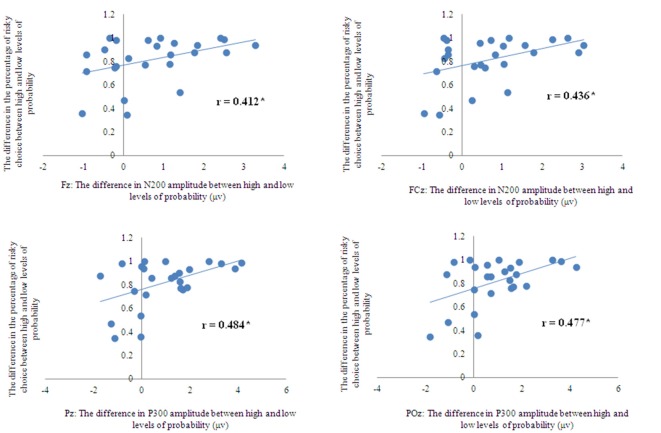
**Correlations between the behavioral data and the ERP data**.

**Figure 5:**
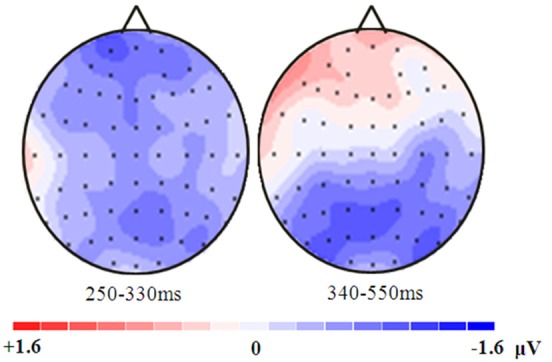
**Topographic distributions of the amplitude differences (low probability minus high probability) of the N2 and P3 in the range of 250–330 ms and 340–550 ms**.

To discern whether the N2 and P3 components represented a process of probability perception, the correlation between the behavioral data and the ERP data was analyzed. The change in the percentage of risky choices between the high level of probability and the low level of probability was calculated for each participant. Accordingly, the changes in the amplitude of the N2 and P3 data were calculated. Then the correlations between the changes in the percentage of risky choices and those changes in the N2 and P3 amplitudes were assessed. Table 2 shows the results of this correlation analysis. Results showed that the change in the amplitude of N2 showed a positive correlation with the change in percentage of risky choice at the Fz and FCz electrodes (Figures 4, 5). The change in the P3 amplitude showed a positive correlation with the change in the percentage of risky choice at the Pz and POz electrodes (Figures 4, 5).

**Table 2:** **Spearman correlation between risky choice and ERP amplitudes**.

	Difference in N2 (250–330 ms) amplitude between high and low levels of probability					
	Fz	FCz	Cz	CPz	Pz	Poz
Difference in percentage between high and low levels of probability	0.412*	0.436*	0.303	0.332	0.277	0.146
	**Difference in P3 (340–550 ms) amplitude between high and low levels of probability**					
	**Fz**	**FCz**	**Cz**	**CPz**	**Pz**	**POz**
Difference in percentage between high and low levels of probability	0.199	0.197	0.247	0.367	0.484*	0.477*

Note: *denotes p < 0.05.

## Discussion

The RT results showed no significant interaction effect between the probability expression and the level of probability. In accordance with the additive factors concept (Sternberg, 1969), the non-significant interaction that observed here indicated that the processing of probability could be divided into two stages: an early one, which was affected by the probability expression, and a later one, which was affected by the level of probability. This result also supported the hypothesis that the probability expression was processed before 100 ms but the level of probability was processed after 200 ms.

Consistent with the levels-of-processing framework advocated by Craik and Lockhart (1972), the ERP results provide further evidence for similar processing of verbal and numerical probabilities. The time sensitivity of the ERP revealed that verbal probability and numerical probability elicited different N1 amplitudes but similar N2 and P3 waveforms.

First, the N1 amplitude (culminating at 100 ms post-onset) at the Oz electrode showed a significant difference between the verbal and numerical probability expressions, suggesting that only the notation character was processed during the early stage of probability processing (100 ms). Level of probability was not found to have any effect during this stage. Previous works have demonstrated that the visual N1 component reflects the operation of a discrimination process within the focus of attention, including pattern recognition and color- and form-based discrimination (Ritter et al., 1983; Vogel and Luck, 2000; Hopf et al., 2002). Consistent with a previous ERP study conducted using Arabic digits, Chinese numerals written in simple form, and Chinese numerals written in complex form served as stimuli (Cao et al., 2010), our finding suggest that the N1 component was reported to be modulated by the presence of the three types of stimuli.

Second, the N2 amplitude elicited by the high level of probability was more negative than that elicited by the low level of probability, suggesting that the N2 component presents an abstract meaning of probability, rather than the denoting a specific expression of probability. Previous works have suggested that the N2 component probably indexes conflict monitoring, with more negative N2 amplitudes indicating higher levels of conflict (Bartholow et al., 2005; Azizian et al., 2006; Folstein and Van Petten, 2008; Mennes et al., 2008). In an ERP study on risky decision making, an enhanced (more negative) N2 amplitude was elicited by a higher level of conflict (Yang et al., 2007; Mennes et al., 2008). In the present study, results showed that the N2 amplitude elicited by the low level of probability was greater than that elicited by the high level of probability. This might indicate that the conflict observed under low-probability conditions was greater than that at medium and high levels of probability. The fact that the N2 amplitude elicited by the verbal probability was more negative than that elicited by the numerical probability at the FCz, Cz, and CPz electrodes may be due to the notation effect found in the N1 component.

Third, the amplitude elicited by the high level of probability was more positive than the one elicited by the low level of probability at the Pz and POz electrodes, indicating that the posterior P3 was involved in the processing of probability level. Previous studies have shown that the P3 is activated when the detection of a stimulus engages the joint operation of attention and working memory (Kok, 2001; Polich, 2007). The low level of probability indicated a higher risk. Consistent with the results of N2, this finding suggested that higher risk required more cognitive capacity or effect to process the probability information.

Finally, a correlation was observed between the P3 component and the percentage of risky choices at the Pz and POz electrodes. The greater P3 amplitude observed at the high level of probability than at the low level of probability was found to lead to a correspondingly higher percentage of risky choices (Figure 4). The correlation between the N2 component and the level of probability was observed at the Fz and FCz electrodes. The more pronounced negative N2 amplitude observed at the low level of probability was found to lead to a lower corresponding percentage of risky choice. The correlation between the behavioral and ERP data provided further evidence that the anterior N2 and posterior P3 components indeed represented a process of probability perception rather than a notation of probability.

Taken together, the RT and the electrophysiological results indicate the existence of two processing stages with identifiable neural substrates, stimulus identification and semantic access, in probability perception during decision making. In the early sensory processing period, the different modes of probability expression (verbal or numerical) affected the ERP waveforms. In the later semantic-associated operations, the different modes of probability expression showed some degree of consistency at any given level of probability.

Ever since numerical probability in its modern form emerged in the 1600s, it has had two faces, frequentist and Bayesian (Fienberg, 2006; Hacking, 2006). Bayesian analysis has found widespread use in a sweeping array of scientific disciplines. Even 12-month-old infants show an ability for pure reasoning in terms of probabilistic inference (Téglás et al., 2011). So far, researchers have not found any evidence for Bayesian inference using verbal probability. The finding of the present work that verbal and numerical probabilities have some level of neuro-consistency may suggest that people may actually make Bayesian inferences during verbal processing.

Turning back to the question put at the beginning, regarding whether the cognitive processing of verbal and numerical probabilities is similar, the current findings provide neural evidence that there is internal consistency between verbal and numerical probabilities. Specifically, they support a view of a two-stage probability processing in which verbal and numerical probabilities are first translated into a common metric and then the semantic meaning of probability (high vs. low) is considered.

## Conflict of Interest Statement

The authors declare that the research was conducted in the absence of any commercial or financial relationships that could be construed as a potential conflict of interest.

## References

[B1] Azizian A. Freitas A. L. Parvaz M. A. Squires N. K. (2006). Beware misleading cues: perceptual similarity modulates the N2/P3 complex. Psychophysiology 43, 253–260. 10.1111/j.1469-8986.2006.00409.x 16805863

[B2] Bartholow B. D. Pearson M. A. Dickter C. L. Sher K. J. Fabiani M. Gratton G. (2005). Strategic control and medial frontal negativity: beyond errors and response conflict. Psychophysiology 42, 33–42. 10.1111/j.1469-8986.2005.00258.x 15720579

[B3] Bonnefon J.-F. Villejoubert G. (2006). Tactful or doubtful? Expectations of politeness explain the severity bias in the interpretation of probability phrases. Psychol. Sci. 17, 747–751. 10.1111/j.1467-9280.2006.01776.x 16984289

[B4] Budescu D. V. Weinberg S. Wallsten T. S. (1988). Decisions based on numerically and verbally expressed uncertainties. J. Exp. Psychol. Hum. Percept. Perform. 14, 281–294. 10.1037/0096-1523.14.2.281

[B5] Cao B. Li F. Li H. (2010). Notation-dependent processing of numerical magnitude: electrophysiological evidence from Chinese numerals. Biol. Psychol. 83, 47–55. 10.1016/j.biopsycho.2009.10.003 19854237

[B6] Clark D. A. (1990). Verbal uncertainty expressions: a critical review of two decades of research. Curr. Psychol. Res. Rev. 9, 203–235. 10.1007/bf02686861

[B7] Craik F. I. M. Lockhart R. S. (1972). Levels of processing: a framework for memory research. J. Verbal Learning Verbal Behav. 11, 671–684. 10.1016/s0022-5371(72)80001-x

[B8] Craik F. I. M. Tulving E. (1975). Depth of processing and the retention of words in episodic memory. J. Exp. Psychol. Gen. 104, 268–294. 10.1037/0096-3445.104.3.268

[B9] Dehaene S. (1996). The organization of brain activations in number comparison: event-related potentials and the additive-factors method. J. Cogn. Neurosci. 8, 47–68. 10.1162/jocn.1996.8.1.47 23972235

[B10] Du X.-L. Liu S.-H. Xu J.-H. Rao L.-L. Jiang C.-M. Li S. (2013). When uncertainty meets life: the effect of animacy on probability expression. Judgm. Decis. Mak. 8, 425–438.

[B11] Fienberg S. E. (2006). When did Bayesian inference become “Bayesian”? Bayesian Anal. 1, 1–40. 10.1214/06-ba101

[B12] Folstein J. R. Van Petten C. (2008). Influence of cognitive control and mismatch on the N2 component of the ERP: a review. Psychophysiology 45, 152–170. 10.1111/j.1469-8986.2007.00602.x 17850238 PMC2365910

[B13] Hacking I. (2006). The Emergence of Probability: A Philosophical Study of Early Ideas about Probability, Induction and Statistical Inference. Cambridge: Cambridge University Press.

[B14] Hopf J.-M. Vogel E. Woodman G. Heinze H.-J. Luck S. J. (2002). Localizing visual discrimination processes in time and space. J. Neurophysiol. 88, 2088–2095. 10.1152/jn.00860.2001 12364530

[B15] Huettel S. A. Song A. W. McCarthy G. (2005). Decision under uncertainty: probabilistic context influences activation of prefrontal and parietal cortices. J. Neurosci. 25, 3304–3311. 10.1523/jneurosci.5070-04.2005 15800185 PMC6724903

[B16] Huettel S. A. Stowe C. J. Gordon E. M. Warner B. T. Platt M. L. (2006). Neural signatures of economic preferences for risk and ambiguity. Neuron 49, 765–775. 10.1016/j.neuron.2006.01.024 16504951

[B17] Jaffe-Katz A. Budescu D. V. Wallsten T. S. (1989). Timed magnitude comparisons ofnumerical and nonnumerical expressions of uncertainty. Mem. Cognit. 17, 249–264. 10.3758/bf03198463 2725262

[B18] Kahneman D. Tversky A. (1982). Variants of uncertainty. Cognition 11, 143–157. 10.1016/0010-0277(82)90023-3 7198958

[B19] Kiefer M. (2005). Repetition-priming modulates category-related effects on event-related potentials: further evidence for multiple cortical semantic systems. J. Cogn. Neurosci. 17, 199–211. 10.1162/0898929053124938 15811233

[B20] Knutson B. Taylor J. Kaufman M. Peterson R. Glover G. (2005). Distributed neural representation of expected value. J. Neurosci. 25, 4806–4812. 10.1523/jneurosci.0642-05.2005 15888656 PMC6724773

[B21] Kok A. (2001). On the utility of P3 amplitude as a measure of processing capacity. Psychophysiology 38, 557–577. 10.1017/s0048577201990559 11352145

[B22] Martin L. E. Potts G. F. (2009). Impulsivity in decision-making: an event-related potential investigation. Pers. Individ. Dif. 46, 303–308. 10.1016/j.paid.2008.10.019 20126284 PMC2663910

[B23] Mennes M. Wouters H. van den Bergh B. Lagae L. Stiers P. (2008). ERP correlates of complex human decision making in a gambling paradigm: detection and resolution of conflict. Psychophysiology 45, 714–720. 10.1111/j.1469-8986.2008.00678.x 18665870

[B24] Mohr P. N. C. Biele G. Heekeren H. R. (2010). Neural processing of risk. J. Neurosci. 30, 6613–6619. 10.1523/JNEUROSCI.0003-10.2010 20463224 PMC6632558

[B25] Paulus M. P. Rogalsky C. Simmons A. Feinstein J. S. Stein M. B. (2003). Increased activation in the right insula during risk-taking decision making is related to harm avoidance and neuroticism. Neuroimage 19, 1439–1448. 10.1016/s1053-8119(03)00251-9 12948701

[B26] Pirtošek Z. Georgijev D. Gregorič-Kramberger M. (2009). Decision making and the brain: neurologists’ view. Interdiscip. Description Complex Syst. 7, 38–53.

[B27] Polich J. (2007). Updating P300: an integrative theory of P3a and P3b. Clin. Neurophysiol. 118, 2128–2148. 10.1016/j.clinph.2007.04.019 17573239 PMC2715154

[B28] Reagan R. T. Mosteller F. Youtz C. (1989). Quantitative meanings of verbal probability expressions. J. Appl. Psychol. 74, 433–442. 10.1037/0021-9010.74.3.433 2737992

[B29] Ritter W. Simson R. Vaughan H. G. (1983). Event-related potential correlates of two stages of information processing in physical and semantic discrimination tasks. Psychophysiology 20, 168–179. 10.1111/j.1469-8986.1983.tb03283.x 6844516

[B30] Rogers R. D. Owen A. M. Middleton H. C. Williams E. J. Pickard J. D. Sahakian B. J.. (1999). Choosing between small, likely rewards and large, unlikely rewards activates inferior and orbital prefrontal cortex. J. Neurosci. 19, 9029–9038. 10516320 10.1523/JNEUROSCI.19-20-09029.1999 PMC6782753

[B31] Shafer G. (1988). “The construction of probability arguments,” in Probability and Inference in the Law of Evidence, eds Tillers P. Green E. D. (Dordrecht: Kluwer Acedemic Publishers), 186–204.

[B32] Smits T. Hoorens V. (2005). How probable is probably? It depends on whom you’re talking about. J. Behav. Decis. Mak. 18, 83–96. 10.1002/bdm.485

[B33] Smith B. W. Mitchell D. G. Hardin M. G. Jazbec S. Fridberg D. Blair R. J.. (2009). Neural substrates of reward magnitude, probability and risk during a wheel of fortune decision-making task. Neuroimage 44, 600–609. 10.1016/j.neuroimage.2008.08.016 18804540 PMC2695942

[B34] Steffen A. Rockstroh B. Wienbruch C. Miller G. A. (2011). Distinct cognitive mechanisms in a gambling task share neural mechanisms. Psychophysiology 48, 1037–1046. 10.1111/j.1469-8986.2011.01177.x 21265864

[B35] Sternberg S. (1969). The discovery of processing stages: extensions of donders’ method. Acta Psychol. 30, 276–315. 10.1016/0001-6918(69)90055-9

[B36] Téglás E. Vul E. Girotto V. Gonzalez M. Tenenbaum J. B. Bonatti L. L. (2011). Pure reasoning in 12-month-old infants as probabilistic inference. Science 332, 1054–1059. 10.1126/science.1196404 21617069

[B37] Teigen K. H. Brun W. (1999). The directionality of verbal probability expressions: Effects on decisions, predictions and probabilistic reasoning. Organ. Behav. Hum. Decis. Process. 80, 155–190. 10.1006/obhd.1999.2857 10527815

[B38] Teigen K. H. Brun W. (2000). Ambiguous probabilities: when does *p* = 0.3 reflect a possibility and when does it express a doubt? J. Behav. Decis. Mak. 13, 345–362. 10.1002/1099-0771(200007/09)13:3<345::aid-bdm358>3.0.co;2-u

[B39] Vogel E. K. Luck S. J. (2000). The visual N1 component as an index of a discrimination process. Psychophysiology 37, 190–203. 10.1111/1469-8986.3720190 10731769

[B40] Wallsten T. S. Budescu D. V. Rapoport A. Zwick R. Forsyth B. (1986). Measuring the vague meanings of probability terms. J. Exp. Psychol. Gen. 115, 348–365. 10.1037/0096-3445.115.4.348

[B41] Wallsten T. S. Budescu D. V. Zwick R. Kemp S. M. (1993). Preferences and reasons for communicating probabilistic information in verbal or numerical terms. B Psychonomic Soc. 31, 135–138. 10.3758/bf03334162

[B42] Weber B. J. Huettel S. A. (2008). The neural substrates of probabilistic and intertemporal decision making. Brain Res. 1234, 104–115. 10.1016/j.brainres.2008.07.105 18710652 PMC2629583

[B44] Windschitl P. D. Wells G. L. (1996). Measuring psychological uncertainty: verbal versus numeric methods. J. Exp. Psychol. Appl. 2, 343–364. 10.1037/1076-898x.2.4.343

[B45] Yang J. Li H. Zhang Y. Qiu J. Zhang Q. (2007). The neural basis of risky decision-making in a blackjack task. Neuroreport 18, 1507–1510. 10.1097/wnr.0b013e3282ef7565 17712284

[B43] Yang J. Zhang Q. (2011). Electrophysiological correlates of decision-making in high-risk versus low-risk conditions of a gambling game. Psychophysiology 48, 1456–1461. 10.1111/j.1469-8986.2011.01202.x 21913928

[B46] Zimmer A. C. (1984). A model for the interpretation of verbal predictions. Int. J. Man Mach. Stud. 20, 121–134. 10.1016/s0020-7373(84)80009-7

